# A Simple and Robust Approach for Evaluation of Antivirals Using a Recombinant Influenza Virus Expressing *Gaussia* Luciferase

**DOI:** 10.3390/v10060325

**Published:** 2018-06-13

**Authors:** Ping Li, Qinghua Cui, Lin Wang, Xiujuan Zhao, Yingying Zhang, Balaji Manicassamy, Yong Yang, Lijun Rong, Ruikun Du

**Affiliations:** 1 College of Pharmacy, Shandong University of Traditional Chinese Medicine, Jinan 250355, China; liping9309@163.com (P.L.); user753951@163.com (Q.C.); lynn942@163.com (L.W.); xiujuan95@163.com (X.Z.); 2 Shandong Provincial Collaborative Innovation Center for Antiviral Traditional Chinese Medicine, Jinan 250355, China; zyy8965@163.com (Y.Z.); yy7204@163.com (Y.Y.); 3 College of Traditional Chinese medicine, Shandong University of Traditional Chinese Medicine, Jinan 250355, China; 4 Department of Microbiology, University of Chicago, Chicago, IL 60637, USA; bmanicassamy@bsd.uchicago.edu (B.M.); 5 Scientific Research Centre, College of Medicine, Shandong University of Traditional Chinese Medicine, Jinan 250355, China; 6 Department of Microbiology and Immunology, College of Medicine, University of Illinois at Chicago, Chicago, IL 60612, USA; lijun@uic.edu (L.R.)

**Keywords:** influenza A virus, *Gaussia* luciferase, antiviral, therapeutics

## Abstract

Influenza A virus (IAV) causes seasonal epidemics and occasional but devastating pandemics, which are major public health concerns. Because the effectiveness of seasonal vaccines is highly variable and the currently available drugs are limited in their efficacy because of the emergence of drug resistance, there is an urgent need to develop novel antivirals. In this study, we characterized a recombinant IAV-carrying *Gaussia* luciferase (Gluc) gene and determined its potential as a tool for evaluating therapeutics. We demonstrated that this recombinant IAV is replication-competent in tissue culture and pathogenic in mice, although it is slightly attenuated compared to the parental virus. Luciferase expression correlated well with virus propagation both in vitro and in vivo, providing a simple measure for viral replication in tissue culture and in mouse lungs. To demonstrate the utility of this virus, ribavirin and oseltamivir phosphate were used to treat the IAV-infected cells and mice, and we observed the dose-dependent inhibition of viral replication by a luciferase assay. Moreover, the decreased luciferase expression in the infected lungs could predict the protective efficacy of antiviral interventions as early as day 2 post virus challenge. In summary, this study provides a new and quantitative approach to evaluate antivirals against IAV.

## 1. Introduction

Influenza A virus (IAV) is a major cause of respiratory infections in humans and is a serious public health threat [1]. Each year, 3 to 5 million people are infected with influenza virus, resulting in up to 500,000 deaths worldwide [2]. Although vaccination is the most effective way to prevent IAV-related diseases for seasonal flu, there is no universal vaccine, and the current vaccination strategies have limitations [3]. Moreover, as a result of widespread associated mutations, resistance to currently available antivirals, including neuraminidase inhibitors (oseltamivir and zanamivir) and M2 inhibitors (amantadine and rimantadine), is increasing [4,5,6,7]. Therefore, there is an urgent need to develop and evaluate vaccines and novel antiviral therapies.

IAVs belong to the Orthomyxoviridae family of enveloped viruses, the genome of which contains eight negative sense, single-stranded viral (v)RNA segments, including PB2, PB1, PA, HA, NP, NA, M, and NS [8]. The vRNAs vary in length from 2341 to 891 nucleotides (nt’s) and are named after the main proteins they encode [9]. However, all eight vRNAs share the same genetic organization: the central coding region, which is in antisense orientation, is flanked at both terminal ends by non-coding regions (NCRs). The NCRs differ in length and in sequence between vRNAs, except for the highly conserved 12- and 13-nt-long sequences at the 3′ and 5′ ends, respectively [9,10].

The reverse genetics of IAVs was originally developed in 1999 and has been well established since then [11,12]. Nowadays, a common method to generate recombinant IAVs is to use the eight-plasmid-based rescue system, of which the core is the “ambisense cassette” within each plasmid [13,14]. This cassette includes both RNA pol I and II sequences, which drive the transcription of vRNAs (pol I) and protein (pol II) expression from the same viral cDNAs [13,14]. The reverse genetics techniques have had an important effect on expanding our knowledge of the molecular biology and pathogenesis of influenza viruses, as well as on developing novel live-attenuated vaccines [8]. In the past decade, influenza reverse genetics systems were further modified by inserting reporter genes such as green fluorescent protein (GFP) and luciferase genes. These reporter IAVs allow for effective tracking of viral infection in vitro and in vivo, enabling a robust quantitative readout. This readout can be used in high-throughput screenings (HTSs) and to assess viral infection easily and reliably without the need for using a secondary assay [15]. However, the complex architecture of the segmented genome confounds the generation of replication-competent reporter IAVs. Firstly, the virus segments are small, and they do not tolerate large insertions. Secondly, insertion of a reporter gene at either end of viral segments disrupts packing signals. Thirdly, most insertions severely attenuate replication and are lost over time [15,16]. 

Multiple strategies have been employed to overcome these hurdles. Manicassamy et al. generated a recombinant IAV carrying a GFP reporter gene fused with NS1 [17]. Although it was attenuated compared with the parental virus, the reporter virus replicated efficiently in mouse lungs and showed pathologic signs in mice [17]. A similar strategy has also been employed to generate IAV expressing *Gaussia* luciferase (Gluc), which was successfully used as a tool for the in vitro study of viral replication, antivirals, and viral host interactions [18]. Avilov et al. utilized a “split-GFP”, in which the 16 C-terminal amino acids of GFP were fused to PB2 and GFP reconstitution occurred in trans-complementing transiently transfected cells [19,20]. This recombinant virus was successfully used to examine intracellular vRNP trafficking [19]. Pena et al. rearranged the *NEP* gene from the NS segment to PB1, followed by inserting reporter genes downstream from either full-length or truncated NS1 [21,22]. The rearranged viruses were further adapted as live-attenuated vaccines or for antiviral drug screening [21,22]. More recently, several bioluminescent reporter IAVs were developed by inserting the luciferase gene into PA, PB2, or NA for in vivo imaging [16,23,24]. These reporter viruses allowed real-time tracking of viral load and dissemination of influenza virus infections in the lungs of mice, facilitating the evaluation of candidate vaccines or novel antiviral interventions on infections in vivo [16,23,24].

In the present study, we examined a recombinant IAV-carrying Gluc reporter gene in NS1 (PR8–NS1–Gluc) in terms of its replication, pathogenicity, and dynamics both in vitro and in vivo and demonstrated that PR8–NS1–Gluc can be used as an easy and reliable means for antiviral evaluation and therapeutic efficacy studies.

## 2. Materials and Methods 

### 2.1. Cells and Plasmids

Human embryonic kidney cell line 293T and Madin-Darby canine kidney (MDCK) epithelial cells were grown in Dulbecco’s modified Eagle’s medium (DMEM; Cellgro, Manassas, VA, USA) supplemented with 10% fetal bovine serum (FBS; Gibco, Carlsbad, CA, USA), 1000 units/mL penicillin, and 100 μg/mL streptomycin (Invitrogen, Carlsbad, CA, USA). Infections were performed in Opti-MEM containing 2 μg/mL *N*-tosyl-l-phenylalanine chloromethyl ketone (TPCK)–trypsin (Sigma-Aldrich, St. Louis, MO, USA). All cells were grown at 37 °C in 5% CO_2_.

The previously described pDZ–NS–GFP plasmid was modified by substituting the *GFP* gene with the *Gluc* gene, generating pDZ–NS–Gluc [25]. The *Gluc* gene was amplified with the primers FR (5′-ggcggtaccgaggccaagcccaccgagaacaacgaagacttc-3′) and RV (5′-gccggatccgtcaccaccggcccccttgatcttg tccacctgg-3′) and inserted into a MCS using *Kpn* I and *Bam*H I. Other IAV rescue plasmids, pDZ–PA, –PB1, –PB2, –NP, –HA, –NA, –M, and –NS1, were used as previously described [25]. All the plasmids were kindly provided by Adolfo Garcia-Sastre (Mt Sinai School of Medicine, New York, NY, USA).

### 2.2. Virus Rescue and Titration

PR8–NS1–Gluc virus (A/Puerto Rico/8/34 background) as well as parental virus PR8 were rescued as previously described [25]. Briefly, 0.5 μg of each of eight pDZ plasmids representing the eight segments of the IAV genome were transfected into 293T/MDCK cocultures using Lipofectamine 2000 (Invitrogen) according to the manufacturer’s instructions. After 48 h, the PR8–NS1–Gluc or IAV–PR8 virus was harvested from the supernatant. After plaque purification, the virus was amplified in 9-day-old embryonated eggs. 

The TCID_50_ values of viral stocks were determined by inoculation of serial 10-fold dilutions of stock virus onto MDCK cells, and the titer was calculated by the Reed–Muench method [26].

### 2.3. Multicycle Replication Assay

In order to perform a multicycle replication assay, MDCK cells growing in 24-well plates were infected by indicated viruses at multiplicities of infection (MOIs) of 0.01 TCID_50_ per cell. After 1 h incubation at 37 °C, the cells were washed, and fresh Opti-MEM containing 2 μg/mL TPCK–trypsin was added. Aliquots were removed at various time points, followed by determination of *Gaussia* luciferase activity and viral titers. 

### 2.4. Gaussia Luciferase Assay

Luciferase assays were performed with a BioLux *Gaussia* Luciferase Assay Kit (NEB, Ipswich, MA, USA) according to the manufacturer’s instructions. In cell culture, 50 μL of culture medium was removed and assayed with 50 μL of luciferase substrate. For animal samples, 20 μL of lung homogenate (appropriate dilution adopted) was added to 50 μL of luciferase substrate, and the relative lighting unit was detected. 

### 2.5. Antiviral Determination

For antiviral determination, infected cells (0.01 MOI) were cultured in Opti-MEM (2 μg/mL TPCK–trypsin) containing increasing concentrations of ribavirin (Sigma-Aldrich) or oseltamivir phosphate (Medchemexpress, Monmouth Junction, NJ, USA). At around 24 h post infection (p.i.), aliquots were removed, and *Gaussia* luciferase activity was determined.

### 2.6. Mouse Infections

Female BALB/c mice (4 to 6 weeks old) were inoculated intranasally with the indicated amount of virus in 30 μL PBS under light isoflurane anesthesia. Body weight was monitored daily. Mice losing 20% of their original body weight were humanely euthanized. At the indicated time, the mice were euthanized, and the lungs were removed for further analysis. Viral load in lung homogenates was determined by both TCID_50_ and the luciferase assay.

For antiviral treatments, mice were treated with either 80 mg/kg/day of ribavirin or 20–50 mg/kg/day of oseltamivir phosphate in PBS, administered by intraperitoneal injection. The treatments were started 2 h before infection and were given twice daily until the end of the experiment. 

All mouse experiments were performed under protocols approved by the Animal Care and Use Committees at Shandong University of Traditional Chinese Medicine, Shandong, China (Approval: SDUTCM-2018007, 5 March 2018). The mice were maintained under specific pathogen-free conditions, and all efforts were made to minimize any suffering as well as the number of animals used in the study.

### 2.7. Haematoxylin and Eosin Staining

The mouse lungs were fixed in 10% buffered formalin, dehydrated, embedded in paraffin, and cut into 5 mm thick sections, followed by staining with haematoxylin and eosin (HE). 

## 3. Results

### 3.1. In Vitro Properties of PR8–NS1–Gluc

**Figure 1:**
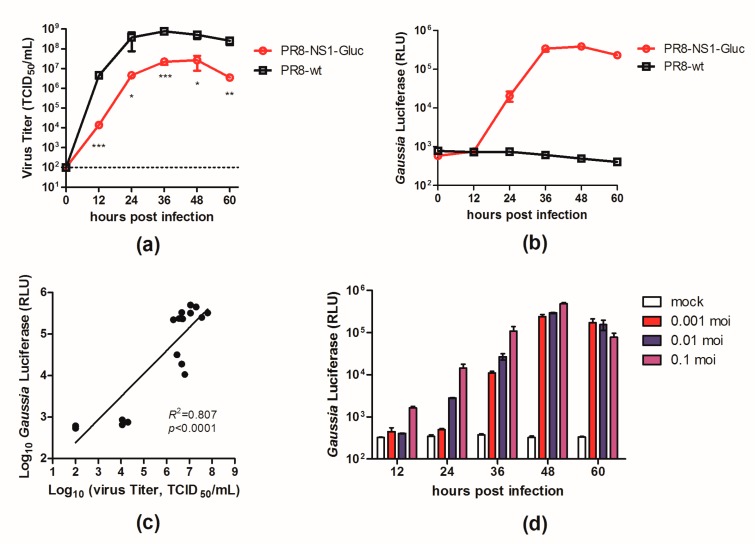
In vitro characterization of PR8–NS1–Gluc. (**a**–**c**) Madin-Darby canine kidney (MDCK) cells were infected with PR8–NS1–Gluc and influenza A virus (IAV)–PR8 at a multiplicity of infection (MOI) of 0.01 and incubated with N-tosyl-L-phenylalanine chloromethyl ketone (TPCK)–trypsin for the indicated times. Aliquotes were removed for determination of (**a**) viral titers and (**b**) *Gaussia* luciferase activity. (**c**) The correlation between supernatant luminescence and infectious virus titers of IAV were fit by linear regression using GraphPad Prism 5 (La Jolla, CA, USA) (*R*^2^ = 0.807, *p* < 0.0001). (**d**) *Gaussia* luciferase signals derived from supernatants of virus-infected cells at MOI of 0.001, 0.01, or 0.1. * *p* < 0.05; ** *p* < 0.01; *** *p* < 0.001.

A luminescent reporter IAV (PR8–NS1–Gluc) was modified from a previously described NS1–GFP virus [17] by replacing the *GFP* gene with the *Gluc* gene. To determine the replication properties of this recombinant virus in tissue culture, we compared the growth kinetics of PR8–NS1–Gluc and the parental PR8 in MDCK cells. MDCK cells were infected with either PR8–NS1–Gluc or PR8 at a MOI of 0.01, and at various time points p.i., the viral titers in the supernatants were quantified. As shown in Figure 1a, PR8–NS1–Gluc showed a slight delay in replication kinetics, with titers reaching 2 × 10^7^ TCID_50_/mL, which was slightly lower than the parental virus (7.5 × 10^8^ TCID_50_/mL).

We also characterized the expression of the luciferase reporter over the viral growth period of PR8–NS1–Gluc in MDCK cells. The supernatants of the infected cells were assayed to detect the luminescent signal; the luciferase signal from PR8–NS1–Gluc increased over 36 h, when both the luciferase signal and viral titer peaked (Figure 1b). The correlation between the luminescence kinetics and the accumulation of infectious virus in the culture supernatants was confirmed (Figure 1c). Additionally, the luciferase signal correlated well with the MOI in the PR8–NS1–Gluc-infected MDCK cells (Figure 1d). 

Collectively, these observations demonstrated that the luciferase activity in the supernatants of the infected cells accurately reflected the proliferation of PR8–NS1–Gluc in vitro.

### 3.2. PR8–NS1–Gluc Virus Is Pathogenic in Mice

**Figure 2:**
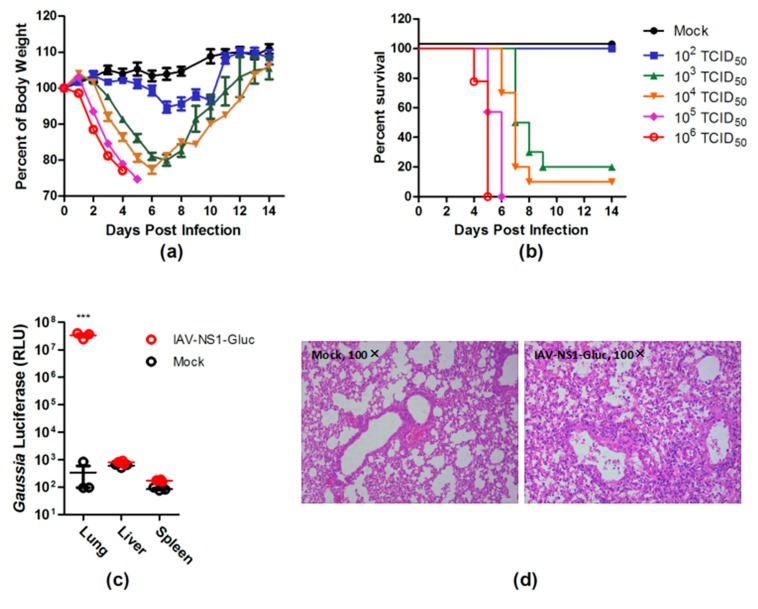
PR8–NS1–Gluc is virulent in mice. (**a**,**b**) BALB/c mice were intranasally inoculated with indicated doses of PR8–NS1–Gluc virus (*n* = 10 in each group). The body weight (**a**) and survival (**b**) were monitored daily. (**c**,**d**) BALB/c mice (*n* = 3) were infected with 10^3^ TCID_50_ of PR8–NS1–Gluc or were mock infected. Three days after infection, the indicated organs were collected, and the levels of luciferase in these organs were determined (**c**). Six days after infection, the lung tissue sections were collected for hematoxylin and eosin staining (**d**). *** *p* < 0.0001.

To assess the virulence of PR8–NS1–Gluc, BALB/c mice were infected intranasally with PR8–NS1–Gluc virus at different doses, and the body-weight loss and survival were monitored daily. Mice that received 10^3^ TCID_50_ or higher doses of the virus showed rapid body-weight loss at and after day 3 p.i. (Figure 2a). The median lethal dose (LD_50_) of PR8–NS1–Gluc was measured to be 610 TCID_50_ on the basis of the survival data (Figure 2b). We conclude that a lethal dose of this virus in this mouse model is easily achievable, suggesting that it can be used as an IAV infectious model in mice. 

IAV infection is an acute infection of the respiratory tract. To determine whether PR8–NS1–Gluc displayed the expected tropism, the indicated tissues of the infected mice were collected for Gluc detection. As expected, the infected lungs showed a roughly 100,000-fold increase in the luciferase signal, which was highly significant statistically (Figure 2c). Further, histopathological analysis revealed evidence of severe pulmonary inflammation, characterized by neutrophil-predominant inflammatory infiltrate, acute alveolar edema, and occasional necrotic debris (Figure 2d).

### 3.3. Dynamics of Luciferase Expression in the Lungs of the Infected Mice

To better characterize the virus in vivo, BALB/c mice were infected with two doses (10^3^ and 10^5^ TCID_50_) of PR8–NS1–Gluc, and the luciferase levels and virus loads in the infected mouse lungs were monitored daily over 6 and 8 days, respectively. 

**Figure 3:**
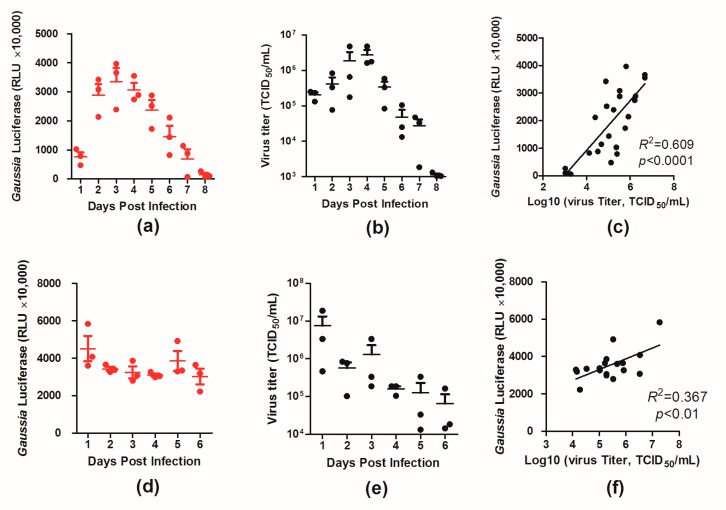
Kinetics of PR8–NS1–Gluc spread and clearance in lungs of BALB/c mice. Mice were intranasally infected with 10^3^ or 10^5^ TCID_50_ of PR8–NS1–Gluc. At the indicated times, lungs were collected from infected animals, and the amounts of luciferase and viral titers were determined. The correlation between the two variants were fit by linear regression using GraphPad Prism 5. (**a**–**c**) Data correspond to the time course for the dose of 10^3^ TCID_50_. (**d**–**f**) Data correspond to the time course for the dose of 10^5^ TCID_50_. The *R*^2^ and *p*-values for the linear regression analysis are indicated on each graph.

For the mice infected with 10^3^ TCID_50_ of the virus, the luciferase level increased and peaked around day 4 p.i., and afterward the luciferase level declined (Figure 3a). A similar trend was observed for virus loads in the infected mouse lungs over time, with a high correlation between the two variables (*R*^2^ = 0.609, *p* < 0.0001; Figure 3b,c). For the mice infected with the higher dose (10^5^ TCID_50_), however, both the luciferase level and virus load in the lungs peaked at day 1 p.i. and started declining afterward (Figure 3d,e). The correlation between the two variables was lower (*R*^2^ = 0.367, *p* < 0.01; Figure 3f), which was likely due to the high doses of the viruses used in infection. These results provide insight into the dynamics of virus infection in mice. 

### 3.4. Evaluation of Antivirals In Vitro

**Figure 4:**
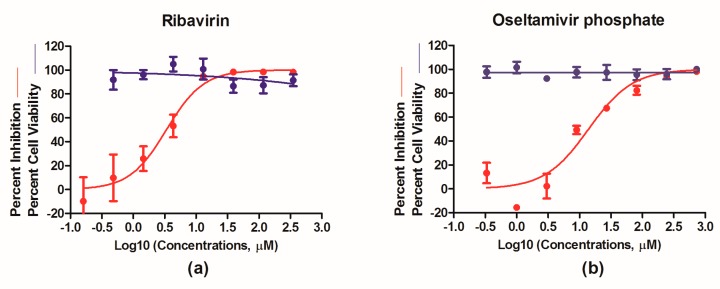
In vitro antiviral determination using PR8–NS1–Gluc as a tool. Madin-Darby canine kidney (MDCK) cells were infected with PR8–NS1–Gluc at a multiplicity of infection (MOI) of 0.01 and incubated with N-tosyl-L-phenylalanine chloromethyl ketone (TPCK)–trypsin as well as increasing concentrations of ribavirin (**a**) or oseltamivir phosphate (**b**) for 24 h. Supernatants were collected for determination of *Gaussia* luciferase activity, and the inhibitory effects were analyzed using GraphPad Prism 5.

We next focused on testing PR8–NS1–Gluc as a robust means to evaluate the efficacy of antiviral therapeutics. Ribavirin, a nucleoside inhibitor, and oseltamivir phosphate, a neuraminidase inhibitor, were tested for their antiviral properties with PR8–NS1–Gluc. Briefly, MDCK cells were infected with PR8–NS1–Gluc at a MOI of 0.01 and incubated for 1 h; unattached viruses were removed, followed by culturing in the absence or presence of increasing concentrations of ribavirin or oseltmivir phosphate, respectively. At 24 h p.i., the luciferase levels in the supernatants were determined. As shown in Figure 4, both ribavirin and oseltamivir phosphate showed antiviral properties in a dose-dependent manner, with IC_50_ values of 3.39 and 14.00 μM, respectively, while no obvious toxicity was observed for either drug at the highest concentrations tested. These results suggest that PR8–NS1–Gluc can be used as a simple means to identify and evaluate antivirals in vitro. 

### 3.5. Evaluation of Antiviral Interventions in a Mouse Model

To demonstrate the feasibility of using PR8–NS1–Gluc for the evaluation of antiviral interventions in mice, female BALB/c mice were infected with 10^3^ TCID_50_ of PR8–NS1–Gluc via intranasal inoculation and treated with ribavirin and oseltamivir phosphate using the infected mice treated with vehicle alone as a control.

**Figure 5:**
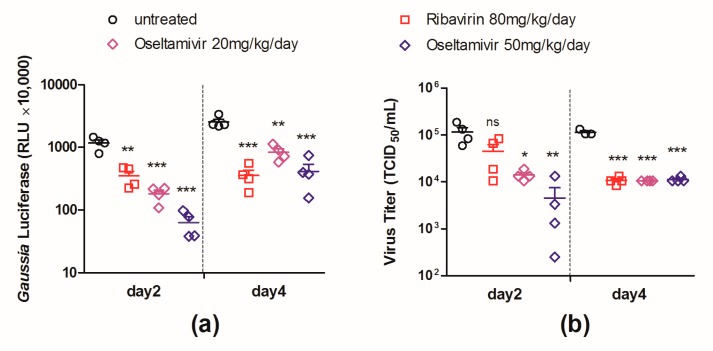
In vivo evaluation of therapeutic interventions using PR8–NS1–Gluc as a tool. Mice were intranasally infected with 10^3^ TCID_50_ of PR8–NS1–Gluc and were treated with indicated drugs by gavage (*n* = 8 in each group). The treatments were started 2 h before infection and were given twice daily until mice were sacrificed. At days 2 and 4 after infection, four mice in each group were dissected and the *Gaussia* luciferase level (**a**) and viral load (**b**), respectively, in infected lungs were determined (ns: no significance; * *p* < 0.05; ** *p* < 0.01; *** *p* < 0.001).

The luciferase levels in the infected lungs of the control group and drug-treated groups were determined at days 2 and 4 p.i., respectively. As shown in Figure 5a, the mice that received treatments of either ribavirin (80 mg/kg/day) or oseltamivir phosphate (50 or 20 mg/kg/day) showed a significant decrease in luciferase expression in the lungs at both time points. We note that the mice treated with the higher dose (50 mg/kg/day) of oseltamivir phosphate had a more drastically reduced luciferase expression than those treated with the lower dose (20 mg/kg/day). 

The viral loads in the lungs of the mice from all groups were also monitored. As shown in Figure 5b, the viral loads in the ribavirin-treated lungs were significantly lower than those in the control group at day 4 p.i. but not at day 2. While viral loads in the oseltamivir phosphate (50 or 20 mg/kg/day) treated lungs showed significant decreases at both day 2 and day 4, the significance at day 2 (*p* = 0.008 or 0.012) was less when compared with the decrease in luciferase levels (*p* = 0.0002 or 0.0004). The results suggest that the decrease in the luciferase level reflected the therapeutic efficacy earlier and more sensitive than the decrease of viral load in mouse lungs.

In order to validate the efficacy of the antiviral treatments, we evaluated the lung index at day 6 after challenge, considering previous studies have shown that a low lung index correlates well with strong protection against virus infection [27]. All antiviral interventions prevented the lung index of the infected mice from increasing (Supplementary Figure S1a). The body weights of the mice in each group were also monitored daily, and body-weight loss was prevented by all antiviral treatments (Supplementary Figure S1b). These data suggest that ribavirin and oseltamivir phosphate at either of the tested doses efficiently restrict virus replication in infected animals.

Together, these data suggest that the luciferase level in the PR8–NS1–Gluc-infected lungs could predict the therapeutic outcome accurately. Moreover, the decrease in the luciferase level could sensitively predict the therapeutic properties as early as day 2 p.i.

## 4. Discussion

Animal models are of great importance in the development of antiviral drugs, with regard to performing preclinical assessments of antiviral candidates. For influenza virus, mice are extensively used as the animal model in these studies [28]. Although mice are not natural hosts for influenza viruses, laboratory strains of mice can be infected with some influenza viruses, including the mice-adapted A/H1N1 strains A/Puerto Rico/8/1934 (PR/8) and A/WSN/1933 (WSN) and the influenza B virus B/Lee/1940 [28].

The establishment of viral reverse genetics and reporter viruses has greatly facilitated the development of animal models as tools for the evaluation of candidate vaccines and therapeutic drugs. So far, luciferase-expressing recombinant viruses have been well demonstrated for herpes simplex virus [29,30], enterovirus 71 [31], Japanese encephalitis virus [32], and Sendai virus [33], as well as for IAV [1,16,24]. Infection of these viruses allows for real-time monitoring in virus replication and dissemination in living animals by noninvasive in vivo imaging [34], facilitating the evaluation of candidate vaccines and therapeutic antibodies. However, the application of noninvasive in vivo imaging, at least in the case of IAV, is still limited because of drawbacks [34]. First, the bioluminescent signal has a limited sensitivity for detection. In most cases, imaging fails to reveal the rapid viral replication shortly after inoculation, although the bioluminescence can be detected when infection is almost peaking or when IAV starts to be cleared [15,35]. Second, manipulation for noninvasive in vivo imaging is complicated [34]. Moreover, the substrate of the most frequently used reporter, *Gaussia* luciferase, among the reporter influenza viruses has flash kinetics and an extremely short imaging window, which makes the data acquisition more difficult and less accurate [36]. 

In this study, a simple and robust mouse model was applied for the evaluation of antiviral therapeutics. To achieve this, a replication-competent recombinant IAV-carrying *Gaussia* luciferase gene (PR8–NS1–Gluc) was generated and further characterized in detail both in vitro and in vivo. 

Our study demonstrated that PR8–NS1–Gluc is replication-competent in MDCK cells and is infectious and pathogenic in mice. Moreover, the expression level of the reporter Gluc could reflect the propagation of the virus in both MDCK cells and the infected lungs. This property allowed us to use Gluc expression to accurately monitor the replication profiles of IAV using Gluc expression with either a sublethal or lethal dose of IAV.

The kinetics of the luciferase level in the infected lungs was monitored and compared to viral loads, giving insight into the dynamics of IAV infection in mice. For the mice infected with the lower dose (10^3^ TCID_50_), the luciferase level and the viral load of the infected lungs were elevated by day 4 and then declined, indicating virus clearance (Figure 3a,b). However, for the mice infected with the higher dose (10^5^ TCID_50_), the luciferase level and viral load peaked earlier, and the dynamics of IAV was less drastic (Figure 3d,e). Our primary experiment showed that oseltamivir phosphate treatment at 50 mg/kg/day could effectively reduce luciferase levels in the lungs for mice infected with 10^3^ TCID_50_ of PR8–NS1–Gluc but showed no efficacy for infection with 10^5^ TCID_50_ (Supplementary Figure S2). These results suggest that the dose of infection is an important factor for animal models aiming to evaluate antiviral interventions.

The feasibility of PR8–NS1–Gluc as a tool for the evaluation of antiviral therapeutics was further validated by testing the anti-influenza drug ribavirin (80 mg/kg/day) and two doses of oseltamivir phosphate (50 and 20 mg/kg/day), all of which showed remarkable therapeutic efficacy (Supplementary Figure S1). On treatment with ribavirin, the monitoring of both the Gluc expression and viral load in the infected lungs produced significant differences between the treated and untreated groups at day 4 p.i. However, at day 2, the viral load failed to reflect the protection by ribavirin, in contrast to the Gluc assay, which demonstrated the protective property significantly (Figure 5). For the mice treated with oseltamivir phosphate, the decrease in both Gluc expression and viral load in the infected lungs could be detected as early as day 2, in a dose-dependent manner. However, the Gluc assay gave a more sensitive measure than the viral load and other traditional measures such as the decreased lung index and mitigated body-weight loss (Figure 5).

In summary, the rapid and sensitive assay developed in this study can significantly reduce the number of animals required, the amount of the candidate therapeutic agents to be administrated, and the duration of the experiment. Therefore, we believe that this approach will be particularly useful as a metric in the primary evaluation of novel therapeutic agents. Moreover, while our study here focused on the evaluation of therapeutics, PR8–NS1–Gluc has potential to be used in basic research on IAV.

## Supplementary Materials

The following are available online at http://www.mdpi.com/1999-4915/10/6/325/s1. Figure S1: in vivo evaluation of therapeutic interventions; Figure S2: evaluation of the therapeutic efficacy of Oseltamivir for infection of different doses.
