# Supplementary material for: A Simple and Robust Approach for Evaluation of Antivirals Using a Recombinant Influenza Virus Expressing Gaussia Luciferase
Source: Viruses. 2018 Jun 13;10(6):325. doi: 10.3390/v10060325 (PMC6024319; doi:10.3390/v10060325)
Supplement: Supplementary file 1 [file viruses-10-00325-s001.pdf]

viruses

MDPI

Article

A Simple and Robust Approach for Evaluation of Antivirals Using A Recombinant Influenza Virus Expressing *Gaussia* Luciferase

Ping Li [1], Qinghua Cui [1,2], Lin Wang [1], Xiujuan Zhao [1], Yingying Zhang [2,3], Balaji Manicassamy [4], Yong Yang [2,3], Lijun Rong [5] and Ruikun Du [1,2,*]

[1] College of Pharmacy, Shandong University of Traditional Chinese Medicine, Jinan 250355, China; liping9309@163.com (P.L.); user753951@163.com (Q.C.); lynn942@163.com (L.W.); xiujuan95@163.com (X.Z.); duzi857@163.com (R.D.)

[2] Shandong Provincial Collaborative Innovation Center for Antiviral Traditional Chinese Medicine, Jinan 250355, China; zyy8965@163.com (Y.Z.); yy7204@163.com (Y.Y.)

[3] Shandong University of Traditional Chinese Medicine, Jinan 250355, China

[4] Department of Microbiology, University of Chicago, Chicago, IL 60637, USA; bmanicassamy@bsd.uchicago.edu (B.M.)

[5] Department of Microbiology and Immunology, College of Medicine, University of Illinois at Chicago, Chicago, IL 60612, USA; lijun@uic.edu (L.R.)

* Correspondence: duzi857@163.com; Tel.: +86-0531-8962-8505

Received: 16 May 2018; Accepted: 11 June 2018; Published: 13 June 2018

Supplementary Materials and methods

S1.1 Evaluation of Lung index

Female BALB/c mice (4 to 6 weeks old) were inoculated intranasally with the indicated amount of virus in 30μl under light isoflurane anesthesia. Body weight was monitored daily. Mice losing 20% of their original body weight were humanely euthanized. At day 6 post-infection, mice were euthanized and the lungs were dissected and weighted, and the lung index was calculated.

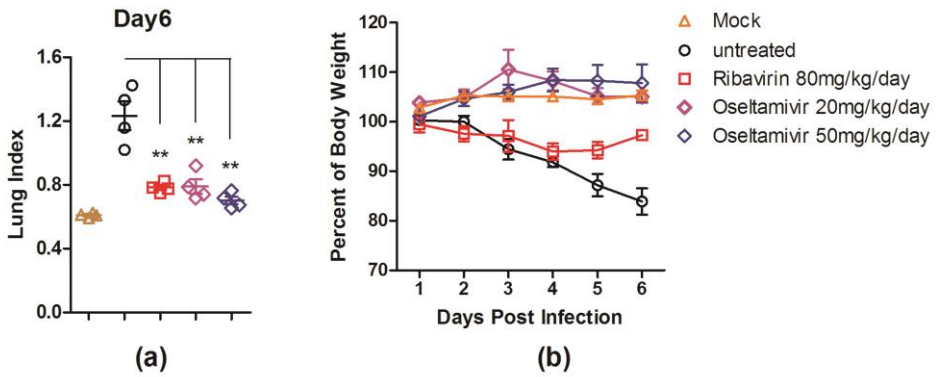

Figure S1. *In vivo* evaluation of therapeutic interventions. Mice were intranasally infected with 10^3 $TCID_{50}$ of PR8-NS1-Gluc, and were treated with indicated drugs by gavage. The treatments were started 2 h before infection and were given twice daily until mice were sacrificed. (a) The lung index of mice were evaluated at day6 after infection. (b) The body weight of mice were monitored daily. **, *p*<0.01.

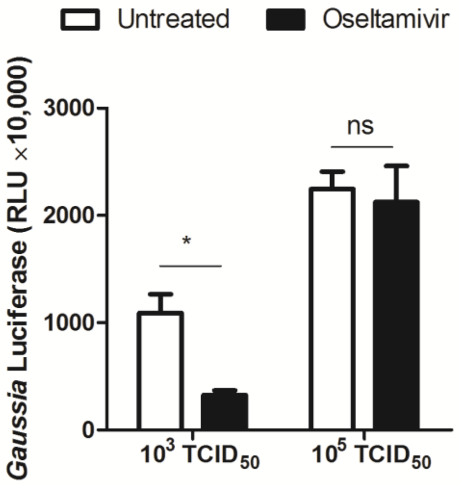

Figure S2. Evaluation of the therapeutic efficacy of Oseltamivir for infection of different doses. Mice were intranasally infected with 10^3 $TCID_{50}$ and 10^5 $TCID_{50}$ of PR8-NS1-Gluc, respectively. Oseltamivir phosphate (50mg/kg/day) were administrated by gavage. The treatments were started 2 h before infection and were given once daily. At day 2 post-infection, the lungs were dissected for luciferase assay. *, $p<0.05$.
